# Health-Related Quality of Life and mental health of families with children and adolescents affected by rare diseases and high disease burden: the perspective of affected children and their siblings

**DOI:** 10.1186/s12887-022-03663-x

**Published:** 2022-10-14

**Authors:** Silke Wiegand-Grefe, Anna Liedtke, Lydia Morgenstern, Antonia Hoff, Anikó Csengoe-Norris, Jessika Johannsen, Jonas Denecke, Claus Barkmann, Benjamin Grolle, Anne Daubmann, Karl Wegscheider, Johannes Boettcher

**Affiliations:** 1 Department of Child and Adolescent Psychiatry, Psychosomatics and Psychotherapy, University Medical Center Hamburg-Eppendorf, Martinistraße 52, 20251 Hamburg, Germany; 2 Department of Pediatrics, University Medical Center Hamburg-Eppendorf, Hamburg, Germany; 3 Department of Medical Biometry and Epidemiology, University Medical Center Hamburg-Eppendorf, Hamburg, Germany

**Keywords:** Health-Related Quality of Life, Mental health, Technologically dependent, Siblings

## Abstract

**Background:**

Advances in genetic and pharmaceutical technology and pediatric care have enabled treatment options for an increasing number of rare diseases in affected children. However, as current treatment options are primarily of palliative nature, the Health-Related Quality of Life (HRQoL) and mental health of this impaired population and their siblings are of increasing importance. Among children and adolescents with rare diseases, those who are technology-dependent carry a high disease burden and are selected as the target population in our study. In a cross-sectional observational design, the children’s HRQoL was assessed with the DISABKIDS (DCGM-37) as well as KIDSCREEN-27, while mental health was assessed with the Strengths and Difficulties Questionnaire (SDQ) by both the affected children, their parents, and siblings.

**Results:**

Results of the study sample were compared to normative data. Affected children scored significantly lower than the norm on almost all HRQoL subscales as reported by parent and child. From the parental perspective, more mental health subscales were significantly impaired compared to the child’s perspective. Siblings showed no impairment in HRQoL as well as significantly fewer behavioral problems and higher prosocial behavior regarding their mental health compared to the norm.

**Conclusion:**

Children and adolescents with rare diseases seem particularly impaired in social and emotional aspects of HRQoL and mental health. Interventions may focus primarily on promoting social skills, fostering prosocial behavior and peer relationships.

## Background

Rare diseases are estimated to affect between 2.4 and 5.0 million people in Germany [1]. The European Union defines these rare diseases by a prevalence of under one in 2000 [2]. Despite the considerable heterogeneity in the clinical presentations of the diseases, the burden on affected families can be quite comparable [3, 4]. The often-delayed diagnosis as well as limited treatment options add to the high level of distress within the family system [5, 6]. The associated physical and emotional strain for the families providing care [7–9] is often detrimental, especially for the patients themselves [10, 11].

Patients with rare diseases face a multitude of challenges, including emotional, cognitive, and physical impairments [12], which can negatively impact their Health-Related Quality of Life (HRQoL) and mental health [13, 14]. HRQoL can be defined as the child’s “perceived well-being in physical, mental and social domains of health […]” [15], whereas mental health can be defined as a “dynamic state of internal equilibrium which enables individuals to use their abilities in harmony with universal values of society. […]” [16]. Considering rare pediatric diseases within the diathesis-stress model [17], the lower HRQoL and mental health of the diseased children can be explained by an interplay of individual vulnerabilities, a high disease burden, and experienced stressful life events.

Previous research shows that chronically ill ventilator-dependent children and adolescents have a decreased self-reported HRQoL compared to healthy peers [18, 19]. Moreover, studies on parent ratings of children requiring long-term home mechanical ventilation show that affected children have a lower HRQoL compared to healthy children [18, 19] and children with chronic diseases [20]. Previous studies could also demonstrate that patients requiring mechanical ventilation have a significantly lower self-reported and parent reported HRQoL compared to pediatric patients with other chronic diseases [21]. Additionally, a recent study found that patients requiring mechanical ventilation had a significantly lowered HRQoL compared to norm values of healthy children, regardless of ventilator usage [22].

Although there is little research evaluating mental health in children requiring mechanical ventilation, a large-scale population study could show that children suffering from a chronic illness had an elevated risk of developing emotional and behavioral problems compared to their healthy peers [23]. Another study found heightened chances of developing a psychiatric illness in all groups of chronically ill children [24]. Previous studies could also show that youths suffering from a chronic disease have a high prevalence of depression and anxiety [25], higher levels of depressive symptoms compared to healthy peers [10], and are more likely to engage in suicidal ideation and self-harm [26]. These mental health problems may persist beyond childhood and adolescence into adulthood [27].

Although the psychosocial needs of siblings of technology-dependent children have been studied qualitatively, to our knowledge, there are no quantitative results on HRQoL and the mental health of affected siblings [14]. However, in an interview-based study, siblings of medical technology-dependent children described an increased number of responsibilities from a young age, either in domestic or medical nature, as well as low mental health, a lack of time for social activities, and tense atmospheres in their homes [28]. While these insights suggest a lowered HRQoL in siblings, studies specifically investigating HRQoL in siblings of chronically ill children have shown mixed results thus far, with some results suggesting equal or even better HRQoL than peers, while others indicate worse HRQoL compared to peers [29].

Since a technological dependency exerts a significant burden on everyday life, it can adversely impact HRQoL [13] and negatively influence mental health [30]. This may particularly apply to children and adolescents affected by rare diseases associated with a specific high need for care. We studied the HRQoL and mental health of the affected children and their siblings using validated instruments. Previous studies investigating the burden of severe chronic diseases have mostly been limited to single disease groups. In contrast, this study aims to examine a study population with a variety of rare diseases, resulting in technological dependency in the progression of the disease. Therefore, the current study addresses the following question: How are HRQoL and mental health of children and adolescents affected by rare diseases with high disease burden and HRQoL and mental health of their siblings distributed?

Four hypotheses were tested in this regard: (1) The HRQoL of children affected by rare diseases and high disease burden is significantly lower compared to norm data of children with various chronic diseases, (2) the mental health of children affected by rare diseases and high disease burden is significantly more impaired in comparison to children and adolescents of population-based norm data, (3) siblings of children affected by rare diseases and high disease burden have significantly lower HRQoL compared to normative data, (4) siblings of children affected by rare diseases and high disease burden have significantly more emotional and behavioral problems in comparison to normative data.

## Methods

### Study design

The study data presented are part of the CHROKODIL project, which evaluated the psychosocial burden of families of children and adolescents with serious physical illness. The current project focused on a group of children with rare diseases and high disease burden, as well as a current or impending technological dependency, using a one-group cross-sectional design. The current study data represents an expansion of the work of Boettcher et al. (2020) [31], who examined the psychosocial distress of parents in the CHROKODIL sample, and the work of Johannsen et al. (2020) [22], who examined the neuromuscular disease subsample regarding the impact of long-term ventilation on psychosocial needs of affected families. Children’s self-report and a parent report were obtained. The study received funding from the Werner Otto Foundation and ethical approval from the Medical Chamber Hamburg (PV 4361).

### Variables and instruments

Sociodemographic and clinical outcomes: Participants were asked to complete a sociodemographic questionnaire about their sex, age, socioeconomic status, and the number of siblings. Clinical outcomes comprised the type and duration of ventilation, as well as the categorization of pediatric conditions according to the recommendations of the German Society of Pneumology and Mechanical Ventilation [32].

Health-related quality of life: The DISABKIDS Chronic Generic Measure-37 (DCGM-37) [33] assesses HRQoL in children and adolescents aged 4 to 16 years. Both the self-report questionnaire (child version), as well as a version for parents were used. Six respective subscales are being measured. Item raw scores are converted into a standardized 0–100 scale, with greater values representing better HRQoL. A total score illustrates overall HRQoL. The DCGM-37 has shown good psychometric properties [34]. The DCGM-37 provides norm data for self-report and parent report ratings of chronically ill children suffering from different chronic diseases [33].

Siblings’ health-related quality of life: The KIDSCREEN-27 [35] was used to assess siblings’ self-reported HRQoL. The instrument consists of 27 items and operationalizes five dimensions. Higher scores indicate greater HRQoL. The KIDSCREEN-27 has shown good psychometric properties [35].

Mental health: The Strengths and Difficulties Questionnaire (SDQ) [36] measures emotional and behavioral strengths and difficulties within five subscales. A total score indicates overall difficulties. In the current study, the German SDQ version for children between the age 3 and 16 years was utilized [37] in parent report and self-report for both diseased children and their siblings. Greater scores indicate more severe problems on every subscale, except for prosocial behavior. The German self-report and parent report versions of the SDQ have shown acceptable internal consistencies [38, 39]. The instrument provides norm data for self-report and parent report ratings of German children and adolescents in the general population [40, 41].

### Sample

Patients included in the study had to be under age 21 suffering from a rare disease with a high disease burden defined as a technological dependency (such as ventilation) and high need for everyday care and support. Patients with a disease progression threatening technological dependency, including the other criteria, were also included. Exclusion criteria were defined as severe physical, mental or cognitive impairments. Parents provided signed informed consent, and if possible, additionally the patients and siblings themselves. It was possible for participants to discontinue their participation in the study at any time.

Regarding the sampling procedure of the CHROKODIL project, reference is made to the previously published articles of the project [22, 31]. In the current study, data on HRQoL and mental health of 62 children and adolescents (38 male, 24 female) were gathered. Parents provided HRQoL and mental health ratings for 44 affected children, and 25 patients provided self-ratings. For seven self-ratings, corresponding parent-ratings were available. In addition, data for 31 siblings were available. Children eight years or older were asked to complete self-ratings on the included questionnaires. Thirty-five (79.5%) of all questionnaires answered by a parent were completed by the mother. All diseases of the participants met the European Commission definition of rare diseases [2].

### Statistics

Group differences between the studied sample and norm reference values were examined with one-sample *t*-tests. Intra-class correlations (ICC) were conducted between patient self-report and parent reports. Cohen’s *d* was calculated to give an estimate of the effect size. Statistical significance was set at *p* ≤ 0.05 (two-tailed). To address possible bias due to missing data, multiple imputation using the Expectation–Maximization approach was used. Statistical analyses were conducted using SPSS Statistics 26.

## Results

### Patient characteristics

Main patient’s characteristics are shown in Table 1. The pediatric diseases included in the sample consisted of central respiratory disorders (9.7%), restrictive ventilatory disorders (80.6%) with neuromuscular disorders (62.9%) as the most frequent conditions, and obstructive ventilatory disorders (9.7%). The chronically diseased sample consisted of more males (61.3%) than females (38.7%). Among the siblings, on the other hand, there were fewer males (32.3%) than females (67.7%). Most children lived with both their mother and father (72.6%). The socioeconomic status was low in 11.3%, moderate in 48.4%, and high in 40.3%.

**Table 1:** Characteristics of the sample

Characteristics	*M*	*SD*
Patient’s age (years)	10.3	4.66
Sibling’s age (years)	13.3	3.20
Mother’s age (years)	41.1	7.13
Father’s age (years)	43.5	6.99
Time on technology (years)	5.9	4.85
	*n*	%
Types of ventilation		
No ventilation	22	35.5
Mask ventilation	21	30.6
Tracheostomy	19	33.9
Duration of ventilation per day		
< than 10 h a day	16	25.8
10 to 24 h a day	16	25.8
24 h a day	8	12.9
Frequency of diagnoses		
RVD	50	80.6
CRRD	6	9.7
OVD	6	9.7

*Note*. *RVD* Restrictive ventilation diseases (Achondroplasia, Autosomal dominant axonal demyelinating neuropathy, Campomelic dysplasia, Centronuclear myopathy, Congenital muscular dystrophy type 1A, Facioscapulohumeral dystrophy, Listeriosis, Mitochondrial cytopathy, Muscular dystrophy type Becker-Kiener, Muscular dystrophy type Duchenne, Myasthenia gravis, Nemaline myopathy, Perinatal asphyxia, Spinal muscular atrophy, Traumatic spinal cord injury, Congenital myopathy), CRRD Central respiratory regulation diseases (Bickerstaff brainstem encephalitis, Dandy-Walker malformation, Joubert syndrome, Leigh syndrome, Ondine Syndrome), *OVD* Obstructive ventilatory disorders (Congenital laryngeal palsy, Congenital tracheomalacia, Mucopolysaccharidosis type I)

### Health-related quality of life

Table 2 presents the mean values of HRQoL according to the DCGM-37 scales for both reports in the study sample. Additionally, a comparison to norm data of children suffering from various chronic conditions is given. On almost all subscales and the total score of the DCGM-37, scores were significantly lower than those of the norm group. This applies to both self-report and parent report, with effect sizes ranging from small to large. However, on the subscale emotion in the parent report and medical treatment for the self-report and parent report, the study sample showed no differences to the norm group. Post-hoc analyses concluded low to moderate agreement between self-reports and parent-reports on HRQoL (ICC = 0.11-0.59).

**Table 2:** Distribution of HRQoL for the self-report and parent report in the DCGM-37 and comparison to norm data of chronically ill children

	Study sample				Norm data^a^		Sample vs. norm data: one sample *t*-test	
	*n*	*M*	[*95%* CI]	*SD*	*M*	*SD*	*p*	*d*
Self-report								
Independence	25	63.5	[55.97; 71.93]	20.42	76.1	18.50	.003	-0.62**
Emotion	25	64.1	[56.05; 72.23]	19.60	73.9	18.92	.004	-0.50**
Social inclusion	25	58.3	[49.88; 66.79]	20.48	74.5	18.14	.001	-0.79***
Social exclusion	25	68.1	[60.02; 76.31]	19.72	84.4	16.24	.001	-0.83***
Physical limitation	25	51.6	[43.21; 60.12]	20.48	73.9	18.92	.001	-1.09***
Medical treatment	16	74.8	[58.84; 78.82]	25.30	72.2	22.62	.690	0.10
DCGM-37 total	25	62.4	[55.55; 69.43]	16.81	76.9	14.80	.001	-0.86***
Parent report								
Independence	44	58.6	[53.40; 63.84]	16.77	76.6	17.25	.001	-1.07***
Emotion	44	66.9	[60.49; 74.39]	22.55	71.6	20.49	.179	-0.21
Social inclusion	44	38.3	[33.46; 43.69]	16.48	74.2	17.65	.001	-2.18***
Social exclusion	44	71.2	[65.57; 77.51]	19.28	80.9	16.79	.002	-0.50**
Physical limitation	44	51.9	[46.08; 58.37]	19.78	70.1	18.32	.001	-0.92***
Medical treatment	30	72.7	[58.17; 73.28]	25.38	69.9	22.15	.537	0.11
DCGM-37 total	44	59.0	[55.51; 62.98]	12.10	74.9	14.55	.001	-1.31***

*Note*. Possible range 0–100. Higher scores indicate better HRQoL. *CI* Confidence interval

^a^Norm data of chronically ill children [33]

^**^p < .01

^***^p < .001 *d* = Cohen’s *d*

### Mental health

Table 3 shows the mean values of emotional and behavioral problems in the sample and a comparison to population-based norm data from Germany. Regarding the children’s self-reports, the sample scored significantly higher only on the peer problems subscale, compared to norm data. In the parent report, the study sample scored significantly higher on the hyperactivity and peer problems subscale, as well as on the total SDQ scale, albeit lower on the prosocial behavior subscale, than norm data, with effect sizes ranging from small to large. Concordance of self- and parent-ratings was found to be poor to medium (ICC = 0.00-0.44).

**Table 3:** Distribution of mental health self-report and parent report ratings in the SDQ and comparison to norm data

	Study sample				Norm data^bc^		Sample vs. norm data: one sample *t*-test	
	*n*	*M*	[*95%* CI]	*SD*	*M*	*SD*	*p*	*d*
Self-report								
Emotional symptoms	25	2.8	[2.01; 3.59]	1.91	2.3	1.9	.204	0.26
Conduct problems	25	1.5	[1.05; 2.07]	1.23	2.0	1.4	.086	-0.40
Hyperactivity	25	2.8	[1.89; 3.86]	2.39	3.7	2.0	.099	-0.38
Peer problems	25	2.7	[2.12; 3.40]	1.56	2.0	1.6	.023	0.45*
Prosocial behavior	25	7.9	[7.18; 8.74]	1.88	7.7	1.7	.496	0.11
Total	25	10.0	[8.15; 11.85]	4.47	10.0	4.6	.999	0.00
Parent report								
Emotional symptoms	44	2.2	[1.62; 2.79]	1.91	2.0	3.05	.482	0.11
Conduct problems	44	1.9	[1.52; 2.44]	1.52	2.2	2.03	.336	-0.19
Hyperactivity	44	4.2	[3.54; 5.00]	2.41	3.2	4.07	.005	0.41**
Peer problems	44	2.4	[1.90; 3.05]	1.90	1.4	2.03	.001	0.53***
Prosocial behavior	44	6.0	[5.34; 6.66]	2.16	8.3	2.03	.001	-1.06***
Total	44	10.9	[9.48; 12.38]	4.76	8.5	6.10	.002	0.50**

*Note.* Possible range for subscales 0–10 and total score 0–40. Higher scores represent lower mental health for all subscales, except prosocial behavior, where lower scores correspond to more difficulties in prosocial behavior. *CI* Confidence interval

^b^Norm data of children and adolescents, self-report [40]

^c^Norm data of children and adolescents, parent report [41]

^*^p < .05

^**^p < .01

^***^p < .001. d = Cohen’s d

### Siblings

Table 4 shows the mean values of HRQoL and mental health in siblings of children with rare diseases and a comparison to norm data. None of the HRQOL scales showed significant differences from the norm. Regarding mental health, siblings scored significantly lower in the subscale conduct problems and significantly higher in prosocial behavior. No other significant differences could be found.

**Table 4:** Distribution of HRQoL and mental health as self-report in siblings and comparison to norm data

	Siblings				Norm data^db^		Sample vs. norm data: one sample *t*-test	
	*n*	*M*	[*95%* CI]	*SD*	*M*	*SD*	*p*	*d*
KIDSCREEN-27								
Emotional symptoms	31	48.4	[45.17; 51.70]	8.90	50.0	10.00	.335	-0.18
Conduct problems	31	50.8	[47.54; 54.07]	8.91	50.0	10.00	.618	0.09
Hyperactivity	31	53.1	[49.33; 56.87]	10.29	50.0	10.00	.104	0.30
Peer problems	31	51.4	[47.78; 54.97]	9.80	50.0	10.00	.440	0.14
Prosocial behavior	31	52.3	[47.85; 56.74]	12.11	50.0	10.00	.300	0.19
SDQ								
Emotional symptoms	31	3.1	[2.16; 4.09]	2.63	2.3	1.9	.089	0.30
Conduct problems	31	1.4	[0.96; 1.88]	1.26	2.0	1.4	.015	-0.48*
Hyperactivity	31	3.9	[3.12; 4.76]	2.24	3.7	2.0	.562	0.09
Peer problems	31	2.5	[1.80; 3.23]	1.95	2.0	1.6	.150	0.26
Prosocial behavior	31	8.5	[7.86; 9.09]	1.67	7.7	1.7	.015	0.47*
Total	31	11.0	[9.03; 12.97]	5.37	10.0	4.6	.308	0.19

*Note.* Possible range for the SDQ subscales 0–10 and total score 0–40. Higher scores represent lower mental health for all subscales, except prosocial behavior, where lower scores correspond to more difficulties in prosocial behavior. *CI* Confidence interval

^b^Norm data of the SDQ children and adolescents, self-report [40]

^d^Norm data of the KIDSCREEN-27 children and adolescents, self-report [35]

^*^p < .05. d = Cohen’s d

## Discussion

Considering that rare diseases can have a major effect on patients and families, it is emphasized that decisions made by health care providers should be grounded on data that come from the affected individuals themselves [42]. Assessing the HRQoL and mental health of affected children and adolescents is therefore especially valuable in tailoring support for this highly stressed group. Consequently, the present study explored HRQoL and mental health of children and adolescents suffering from rare diseases resulting in technological dependency and their siblings.

Findings indicate that children with rare diseases have significantly lower self- and parental perceptions of global HRQoL in comparison to norm data of children and adolescents with various chronic diseases, which is in line with previous research [18, 43]. These results thus confirm our first hypothesis within the diathesis-stress model [17]. Although the study group had significantly lower scores in most of the subscales, this pattern could not be found for the treatment subscale, which assesses the perceived impact of receiving medical treatment. Interestingly, no significant difference for both self and parental perceptions could be found in this subscale. One interpretation of this finding could be the vital role of treatment for the survival of these technology-dependent children and adolescents with a rare disease, hence it might not be viewed negatively [44]. It is likely that parents view their older children as more accustomed to the medical treatment, which may also have a positive impact on this subscale. Albeit a significant difference between the study group and the norm group was found in the self-reported emotional dimension of HRQoL, no difference was found in the parent report. While the emotional impact of the disease may be highly relevant for the affected children and adolescents, this dimension may be rather difficult for the parents to recreate fully. This is in line with prior findings, which state that agreement on HRQoL ratings is generally lower in non-observable subscales [45]. As previous research already stated [18], patients may become more aware of their situation with growing age and therefore perceive a higher physical limitation. Our research suggests that there is poor to moderate agreement between parent and child ratings concerning HRQoL. However, parents perceived their children’s HRQoL as generally lower than the children themselves, which is in concordance with previous research [18, 44]. Affected children might focus more on their individual abilities and thus rate their HRQoL as higher compared to their parents, whose lower ratings may be linked to their overprotectiveness and concerns [3].

With regards to emotional and behavioral problems, the parent reported overall difficulties score was significantly higher in the study sample compared to peers, which is consistent with past research on the mental health of children with chronic diseases [25, 27]. Greater levels of internalizing and externalizing problems reported in former research on chronically physically ill youth [10, 23] were only partially reflected by our data. This might be a consequence of using rather unspecific broad-band scales, wherein the context of our study, some sensitivity might get lost, as suggested in previous research for samples with a low risk of mental disease [46]. However, considering that our sample is classified as high-risk for psychiatric illnesses, the use of the broader SDQ subscales was not shown to be helpful.

A differentiated view on the mental health subscales reveals that parents rated their children’s hyperactivity as significantly higher in comparison to norm data of German children, which is consistent with previous studies [23, 24]. Furthermore, the study group presents difficulties in social subscales of mental health. Even though the patients did not perceive their prosocial behavior to be significantly different compared to other children, their parents did. Since pediatric chronic conditions have been linked to social deficits [47], it can be assumed that the patients in the present study have compromised social skills. This is in line with previous research indicating that permanent dependency on medical technological devices creates significant barriers in social life [48, 49] and is linked to lowered self-esteem due to social exclusion, as it is experienced by some ventilator-dependent children [50]. This suggestion is underlined by the fact that not only the parents but also the affected children gave significantly higher ratings on the subscale peer problems compared to norm data. Difficulties in social interaction have already been addressed regarding HRQoL in the present study and may put pediatric patients at risk for having peer problems. Thus, children with rare diseases may not have the chance to develop appropriate social skills for forming healthy relationships with same-aged youths. Apparently, as reflected by our data, the second hypothesis especially applies to social aspects of mental health. As mentioned before, neither the use of the rather unspecific broad-band scales nor the previous gender-specific findings seem to be the case for this highly impaired sample. With respect to emotional and behavioral problems, self-report and parent-ratings on the total difficulties score and the corresponding subscales had poor concordance.

Although research to date on HRQoL of siblings of children with chronic diseases has suggested that some siblings may have lowered HRQoL due to the impact of the disease on the home environment [51], this could not be found in our study sample. Instead, it was shown that siblings of children with rare diseases have comparable HRQoL to normative control data. In addition, siblings reported neither impairment in their HRQoL, nor their mental health, even indicating fewer conduct problems and more prosocial behavior than the healthy norm. One explanation for this finding could be that siblings have a more positive view of what it means to be healthy because they experience their sister's or brother's illness as a daily reference [52], or that taking on more responsibilities from a younger age [28] teaches them to act more prosocial. Moreover, there is evidence that having a chronically ill brother or sister may positively affect the healthy sibling, including a more caring and warm personality [53]. However, due to the overall mixed results on HRQoL in siblings of children with chronic diseases, it might still be helpful to offer targeted interventions to improve the well-being of siblings. For instance, special programs or leisure camps aimed at adjusting to having a sibling with a chronic illness can help build knowledge and self-esteem [51]. These results indicate that the third and fourth hypotheses could not be confirmed.

### Strengths and limitations

Within the study the following limitations should be considered. Due to the long study duration and demands of data collection on patients, this study is based on a small sample size, limiting statistical power and generalizability and therefore results should be evaluated with care. However, recruitment of this study group is particularly challenging due to the severity of the diseases. Although the heterogeneity of rare diseases might be a limitation, there was one consistent feature, meeting the definition of a rare disease resulting in technological dependency in the progression of the disease. Thus, the study sample does represent severe somatic illnesses, that are linked to a particularly high level of care and disease management.

Recruitment of the patients took place in two institutions in Germany. Therefore, transferring the results to countries with other health care systems should be considered with precaution. Additionally, due to the fact that only a part of the identified families could be included within the CHROKODIL project, a selection bias cannot be ruled out. Regardless of these constraints, the findings of the present study deserve to be considered as relevant data indicating the importance of psychosocial support for children with severe rare diseases and their siblings. Thus, somatic treatment should ideally go hand in hand with individually tailored psychosocial interventions.

## Conclusions

The results contribute to the existent research by demonstrating that HRQoL and mental health in children and adolescents with rare diseases resulting in technological dependency in the progression of the disease may be negatively impacted, especially regarding social and emotional aspects with medium to high effects. Therefore, social deficits and peer problems may best be targeted in specific programs that teach social skills and foster prosocial behavior and peer relationships, reducing loneliness by improving social acceptance. Regarding the assessment of HRQoL and mental health, both self-report and parent reports should be obtained whenever possible. Finally, this study highlights the importance of the psychosocial assessment of siblings in families with a child diagnosed with a rare disease.

## Data Availability

The datasets generated during the current study are available from the corresponding author (JB) on reasonable request.

## Abbreviation

HRQOL: Health-related Quality of Life

## References

[1] Mundlos C (2017). Bessere Versorgungsstrukturen für seltene Erkrankungen. Monatsschr Kinderheilkd..

[2] European Commission. Rare diseases: a major unmmet medical need. Publications Office of the EU. 2017.

[3] Pelentsov LJ, Fielder AL, Laws TA, Esterman AJ (2016). The supportive care needs of parents with a child with a rare disease: results of an online survey. BMC Fam Pract.

[4] Anderson M, Elliott EJ, Zurynski Y (2013). Australian families living with rare disease: experiences of diagnosis, health services use and needs for psychosocial support. Orphanet J Rare Dis.

[5] Zurynski Y, Deverell M, Dalkeith T, Johnson S, Christodoulou J, Leonard H (2017). Australian children living with rare diseases: experiences of diagnosis and perceived consequences of diagnostic delays. Orphanet J Rare Dis.

[6] van Oers HA, Haverman L, Limperg PF, van Dijk-Lokkart EM, Maurice-Stam H, Grootenhuis MA (2014). Anxiety and depression in mothers and fathers of a chronically ill child. Matern Child Health J.

[7] Smith J, Cheater F, Bekker H (2015). Parents’ experiences of living with a child with a long-term condition: a rapid structured review of the literature. Health Expect.

[8] Boettcher J, Boettcher M, Wiegand-Grefe S, Zapf H (2021). Being the pillar for children with rare diseases—a systematic review on parental quality of life. Int J Environ Res Public Health.

[9] Boettcher J, Zapf H, Fuerboeter M, Nazarian R, Reinshagen K, Wiegand-Grefe S (2021). Perceived mental health in parents of children with rare congenital surgical diseases: a double ABCX model considering gender. Orphanet J Rare Dis.

[10] Pinquart M, Shen Y (2011). Behavior problems in children and adolescents with chronic physical illness: a meta-analysis. J Pediatr Psychol.

[11] Fuerboeter M, Boettcher J, Barkmann C, Zapf H, Nazarian R, Wiegand-Grefe S (2021). Quality of life and mental health of children with rare congenital surgical diseases and their parents during the COVID-19 pandemic. Orphanet J Rare Dis.

[12] von der Lippe C, Diesen PS, Feragen KB (2017). Living with a rare disorder: a systematic review of the qualitative literature. Mol Genet Genomic Med.

[13] Cohen JS, Biesecker BB (2010). Quality of life in rare genetic conditions: A systematic review of the literature. Am J Med Genet A.

[14] Mesman GR, Kuo DZ, Carroll JL, Ward WL (2013). The impact of technology dependence on children and their families. J Pediatr Health Care.

[15] Hays RD, Reeve BB, Killewo J, Heggenhougen HK, Quah SR (2010). Measurement and modeling of health-related quality of life. Epidemiology and Demography in Public Health.

[16] Galderisi S, Heinz A, Kastrup M, Beezhold J, Sartorius N (2015). Toward a new definition of mental health. World Psychiatry.

[17] Burke P, Elliott M (1999). Depression in pediatric chronic illness: a diathesis-stress model. Psychosomatics.

[18] Gonzalez R, Bustinza A, Fernandez SN, Garcia M, Rodriguez S, Garcia-Teresa MA (2017). Quality of life in home-ventilated children and their families. Eur J Pediatr.

[19] Bojanić K, Grizelj R, Vuković J, Omerza L, Grubić M, Ćaleta T (2018). Health-related quality of life in children and adolescents with congenital diaphragmatic hernia: a cross-sectional study. Health Qual Life Outcomes..

[20] Graham RJ, Rodday AM, Parsons SK (2014). Family-centered assessment and function for children with chronic mechanical respiratory support. J Pediatr Health Care.

[21] Wei Y, Speechley K, Campbell C (2015). Health-Related Quality of Life in children with duchenne muscular dystrophy: a review. J Neuromuscul Dis.

[22] Johannsen J, Fuhrmann L, Grolle B, Morgenstern L, Wiegand-Grefe S, Denecke J (2020). The impact of long-term ventilator-use on Health-Related Quality Of Life and the mental health of children with neuromuscular diseases and their families: need for a revised perspective?. Health Qual Life Outcomes.

[23] Hysing M, Elgen I, Gillberg C, Lie SA, Lundervold AJ (2007). Chronic physical illness and mental health in children. results from a large-scale population study. J Child Psychol Psychiatr.

[24] Hysing M, Elgen I, Gillberg C, Lundervold AJ (2009). Emotional and behavioural problems in subgroups of children with chronic illness: Results from a large-scale population study. Child Care Health Dev.

[25] Buchberger B, Huppertz H, Krabbe L, Lux B, Mattivi JT, Siafarikas A (2016). Symptoms of depression and anxiety in youth with type 1 diabetes: a systematic review and meta-analysis. Psychoneuroendocrinol.

[26] Barnes AJ, Eisenberg ME, Resnick MD (2010). Suicide and self-injury among children and youth with chronic health conditions. Pediatr.

[27] Secinti E, Thompson EJ, Richards M, Gaysina D (2017). Research review: Childhood chronic physical illness and adult emotional health - A systematic review and meta-analysis. J Child Psychol Psychiatry.

[28] Heaton J, Noyes J, Sloper P, Shah R (2005). Families’ experiences of caring for technology-dependent children: a temporal perspective. Health Soc Care Community.

[29] Rana P, Mishra D (2015). Quality of life of unaffected siblings of children with chronic neurological disorders. Indian J Pediatr.

[30] Nunn R (2017). “It’s not all in my head!”-The complex relationship between rare diseases and mental health problems. Orphanet J Rare Dis.

[31] Boettcher J, Denecke J, Barkmann C, Wiegand-Grefe S (2020). Quality of life and mental health in mothers and fathers caring for children and adolescents with rare diseases requiring long-term mechanical ventilation. Int J Environ Res Public Health.

[32] Windisch W, Dreher M, Geiseler J, Siemon K, Brambring J, Dellweg D (2017). Guidelines for non-invasive and invasive home mechanical ventilation for treatment of chronic respiratory failure - update 2017. Pneumologie.

[33] DISABKIDS Group. The DISABKIDS questionnaires-quality of life questionnaires for children with chronic conditions. Lengerich: Pabst Science Publishers; 2006.

[34] Simeoni MC, Schmidt S, Muehlan H, Debensason D, Bullinger M, DISABKIDS Group (2007). Field testing of a European quality of life instrument for children and adolescents with chronic conditions: the 37-item DISABKIDS chronic generic module. Qual Life Res.

[35] Ravens-Sieberer U, Schmidt S, Gosch A, Erhart M, Petersen C, Bullinger M (2007). Measuring subjective health in children and adolescents: results of the European KIDSCREEN/DISABKIDS Project. Psychosoc Med.

[36] Goodman R (1997). The strengths and difficulties questionnaire: a research note. J Child Psychol Psychiatry.

[37] Klasen H, Werner W, Rothenberg A, Goodman R (2003). Die deutsche fassung des strengths and difficulties questionnaire (SDQ-Deu) – übersicht und bewertung erster validierungs- und normierungsbefunde. Prax Kinderpsychol Kinderpsychiatr.

[38] Lohbeck A, Schultheiß J, Petermann F, Petermann U (2015). Die Deutsche Selbstbeurteilungsversion des Strengths and Difficulties Questionnaire (SDQ-Deu-S). Diagnostica..

[39] Woerner W, Becker A, Friedrich C, Klasen H, Goodman R, Rothenberger A (2002). Normierung und evaluation der deutschen elternversion des Strengths and Difficulties Questionnaire (SDQ): Ergebnisse einer repräsentativen felderhebung. Z Kinder Jugendpsychiatr Psychother.

[40] Becker A, Wang B, Kunze B, Otto C, Schlack R, Hölling H (2018). Normative data of the self-report version of the German strengths and difficulties questionnaire in an epidemiological setting. Z Kinder Jugendpsychiatr Psychother.

[41] Hölling H, Schlack R, Petermann F, Ravens-Sieberer U, Mauz E (2014). Psychische auffälligkeiten und psychosoziale beeinträchtigungen bei Kindern und Jugendlichen im Alter von 3 bis 17 Jahren in Deutschland - Prävalenz und zeitliche Trends zu 2 Erhebungszeitpunkten (2003–2006 und 2009–2012): Ergebnisse der KiGGS-Studie - Er. Bundesgesundheitsblatt Gesundheitsforschung Gesundheitsschutz.

[42] Hytiris M, Johnston D, Mullen S, Smyth A, Dougan E, Rodie M (2021). Experience of health care at a reference centre as reported by patients and parents of children with rare conditions. Orphanet J Rare Dis.

[43] Cramm JM, Strating MM, Sonneveld HM, Nieboer AP (2013). The longitudinal relationship between satisfaction with transitional care and social and emotional quality of life among chronically ill adolescents. Appl Res Qual Life..

[44] Mattson J, Lunnelie J, Löfholm T, Andersson ES, Aune RE, Björling G (2022). Quality of life in children with home mechanical ventilation - a scoping review. SAGE Open Nurs.

[45] Qadeer RA, Ferro MA (2018). Child-parent agreement on health-related quality of life in children with newly diagnosed chronic health conditions: a longitudinal study. Int J Adolesc Youth.

[46] Goodman A, Lamping DL, Ploubidis GB (2010). When to use broader internalising and externalising subscales instead of the hypothesised five subscales on the Strengths and Difficulties Questionnaire (SDQ): Data from British parents, teachers and children. J Abnorm Child Psychol.

[47] Pinquart M, Teubert D (2012). Academic, physical, and social functioning of children and adolescents with chronic physical illness: a meta-analysis. J Pediatr Psychol.

[48] Carnevale FA, Alexander E, Davis M, Rennick J, Troini R (2006). Daily living with distress and enrichment: the moral experience of families with ventilator-assisted children at home. Pediatr.

[49] Sarvey SI (2008). Living with a machine: the experience of the child who is ventilator dependent. Issues Ment Health Nurs.

[50] Noyes J (2006). Health and quality of life of ventilator-dependent children. J Adv Nurs.

[51] Limbers CA, Skipper S (2014). Health-related quality of life measurement in siblings of children with physical chronic illness: a systematic review. Fam Syst Health..

[52] Havermans T, Wuytack L, Deboel J, Tijtgat A, Malfroot A, de Boeck C (2011). Siblings of children with cystic fibrosis: quality of life and the impact of illness. Child Care Health Dev.

[53] Barlow JH, Ellard DR (2006). The psychosocial well-being of children with chronic disease, their parents and siblings: An overview of the research evidence base. Child Care Health Dev.

